# Effects of two weeks of metformin treatment on whole-body glycocalyx barrier properties in db/db mice

**DOI:** 10.1186/1475-2840-12-175

**Published:** 2013-12-05

**Authors:** Bart JM Eskens, Coert J Zuurbier, Judith van Haare, Hans Vink, Jurgen WGE van Teeffelen

**Affiliations:** 1 Department of Physiology, Cardiovascular Research Institute Maastricht (CARIM), Maastricht University, PO Box 616, 6200, MD Maastricht, The Netherlands; 2 Department of Anesthesiology, Academic Medical Center, University of Amsterdam, Amsterdam, The Netherlands

**Keywords:** Glycocalyx, Metformin, db/db, Diabetes

## Abstract

**Background:**

The anti-diabetic drug metformin has been demonstrated to exert a protective effect against vascular complications in diabetes independent of its glucose lowering action. Since the endothelial glycocalyx has been indicated to have important vasculoprotective properties and to be vulnerable to degradation by hyperglycemic conditions, we evaluated in the current study the effect of short-term metformin treatment on whole-body glycocalyx barrier properties in a mouse model of non-insulin dependent diabetes mellitus (db/db mouse).

**Methods:**

Glycocalyx barrier properties were measured in an acute experiment in three groups of mice: 1) db/db mice without treatment serving as controls, 2) db/db mice which received metformin for two weeks in the drinking water serving as experimental group, and 3) C57Bl/6 mice serving as reference group. Animals were put under anesthesia (ketamine, medetomidine, and atropine) and carotid artery blood pressure was continuously monitored. To probe the glycocalyx a mixture of the tracers FITC-labeled 70 kDa dextrans (Dex70) or fluorescein-labeled red blood cells (RBCs) versus Texas Red-labeled 40 kDa dextrans (Dex40) was infused and blood samples subsequently collected for 30 min to determine the initial vascular distribution volume and clearance of these tracers. Urine was collected and dry-to-wet weight of heart and kidney were determined after the experiment. Group differences were tested using unpaired t-tests.

**Results:**

Metformin treatment did not affect body weight, fasting blood glucose and arterial blood pressure. Compared to C57Bl/6 mice, db/db mice showed a diminished initial exclusion and increased vascular clearance of Dex70 versus Dex40 (P < 0.05), and both were improved by the metformin treatment (P < 0.05). While urine production was higher in the db/db mice compared to C57Bl/6 (P < 0.05), heart and kidney of the metformin treated animals showed comparable dry-to-wet weights compared to the C57Bl/6 mice.

**Conclusions:**

Two weeks of metformin in the drinking water is associated with an improvement in glycocalyx barrier properties in db/db mice, as evidence by an enhanced exclusion and retention of 70 kDa dextrans in the vasculature. In addition, metformin improved hydration of heart and kidney. Previous reported cardiovascular benefits of metformin may well involve an improvement of the endothelial glycocalyx.

## Background

Metformin (dimethylbiguanide) is an orally administered insulin-sensitizing drug used to lower blood glucose concentrations in patients with non-insulin dependent diabetes mellitus (NIDDM) [1,2]. Compared with other biguanides, metformin expresses the best balance of potent activity and low toxicity. It is the most popular anti-diabetic drug in the United States and one of the most prescribed drugs in this country. The insulin-sensitizing effect of metformin has been suggested to be the result of many actions, including an increased insulin-mediated glucose disposal, increased translocation of glucose transporters, suppressed hepatic glucose output, increased intestinal glucose use and decreased fatty-acid oxidation [1,3,4]. In addition to its glucose lowering effects, metformin has been found to exhibit beneficial cardiovascular effects in patients with NIDDM [5-7]. A major benefit appears to be the alleviation of endothelial dysfunction [8-10], and metformin was for example demonstrated to increase nitric oxide (NO) bioavailability [11]. As a result, it is considered that metformin has vasculoprotective properties [12], but the mechanisms behind these actions are not completely understood at the moment.

The endothelial glycocalyx is the polysaccharide rich, gel-like layer located at the luminal side of all blood vessels, which has been demonstrated the last decades to be a gatekeeper of the vascular wall by regulating many aspects of endothelial function [13-15]. In various experimental models, enzymatic glycocalyx breakdown was shown to reduce shear stress dependent NO production [16], to increase leukocyte and platelet adherence to the endothelium [17,18], and to increase fluid and protein leakage across the vasculature [19,20]. Recent studies from our laboratory showed that hyperglycemic conditions in humans (i.e., 6 h of a hyperglycemic clamp in healthy controls [21], type 1 [22] and 2 diabetics [23]) were associated with reduced glycocalyx dimensions and/or an increased release of glycocalyx constituents in plasma. These indications that high glucose levels induce the loss of glycocalyx barrier properties in humans were preceded by an experimental study in mice. This study showed that the vascular retention of 70 kDa dextrans, considered as a plasma tracer whose access into the glycocalyx is significantly limited, was impaired during acute hyperglycemia in healthy animals and during chronic hyperglycemia in the db/db model of NIDDM [24]. The loss of glycocalyx barrier properties was accompanied by a disturbed fluid balance, as reflected by dehydration of heart and kidney, and significant increases in systematic hematocrit and urine production [24].

Given the vasculoprotective effects of metformin on the one hand, and the indicated role of the glycocalyx in orchestrating vascular wall homeostasis on the other hand, we hypothesize that metformin may improve glycocalyx properties in NIDDM. We tested this hypothesis in the current study in the db/db mouse model of NIDDM by determining whole-body volume distribution of circulating plasma and 70 kDa dextrans versus 40 kDa dextrans and the vascular retention of both dextrans [25] after administration of metformin in the drinking water for 2 weeks. In addition, the effect of metformin on the whole-body fluid balance was evaluated by measurements of hematocrit, urine production, and kidney and heart hydration [24]. The data of the current study indicate that short-term metformin treatment in a mouse model of NIDDM is associated with improved glycocalyx barrier properties and tissue hydration.

## Methods

### Animals & treatments

All procedures were in accordance with requirements of the Animal Ethics Care and Use committee of the Academic Medical Center in Amsterdam. Experiments were performed on male db/db and C57Bl/6 mice which were obtained from Harlan (Horst, the Netherlands) at an age of 5 weeks. After arrival from the supplier, db/db mice (n = 20) received standard chow and water ad libitum for two weeks. Then, after an overnight fast db/db mice were weighed and a blood sample collected via puncture of the saphenous vein for determination of blood glucose concentration with a glucose meter (Ascensia Contour). Db/db mice were subsequently divided into two groups: the experimental group (n = 10) received metformin in their drinking water (0.33 mg/ml) for two weeks until the acute experiment, while the control group (n = 10) maintained receiving normal drinking water for this period. For n = 6 animals in each group, daily intake of water was estimated by weighing of the water bottle every day; as a consequence, these animals were housed in groups of two mice per cage. Midway the treatment period, body weights and fasting blood glucose were measured for the second time in the db/db mice. C57Bl/6 mice (n = 10) receiving normal drinking water served as reference group; no measurements were performed in these animals until the acute experiment.

### Acute experiment

Mice were overnight fasted. Preparation of the animals on the day of experiment was as described previously [25]. Briefly, the mice were anesthetized with ketamine (125 mg/kg), medetomidine (0.2 mg/kg), and atropine (0.5 mg/kg). A tracheotomy was performed and mechanical ventilation was started by connecting the trachea tube to a pressure-controlled ventilator (SAR-830/P; CWE). Animals were ventilated with a gas mixture of 1:1 O_2_:N_2_. Respiration was set at 90 breaths/min with a peak inspiratory pressure of 18 cmH_2_O and a positive end-expiratory pressure of 2 cmH_2_O. Depth of anesthesia was maintained according to stability of blood pressure and lack of toe pinch reflex by continuous i.p. infusion at a rate of 10 ml/kg/h of ketamine (3.5 mg/ml), medetomidine (20 μg/ml) and atropine (7.5 μg/ml). The carotid artery and jugular vein were cannulated for monitoring blood pressure and heart rate, and for infusion purposes, respectively. Temperature was controlled at 37˚C using rectal temperature monitoring, a temperature-controlled heating pad and an infrared lamp. After instrumentation, an equilibration period of 30 min was allowed. A blood sample was taken from the tail for measurement of blood glucose.

To delineate the glycocalyx barrier properties, a bolus injection of two distinct tracers was administered, and their dilution in blood measured for 30 min [24-27]. In each mouse, the distribution volume of either FITC-labelled 70 kDa dextrans (Dex70; Sigma-Aldrich) (n = 5 per group) or circulating plasma (n = 5 per group) as glycocalyx-hindered tracer was compared to that of simultaneously infused Texas Red-labelled 40 kDa dextrans (Dex40; Invitrogen-Molecular Probes), which are considered to have unlimited access to the entire intraluminal volume [25,28]. 0.1 ml of dextran mix (2.5 mg/ml Dex70 + 10 mg/ml Dex40 in phosphate-buffered saline) was manually infused in the jugular vein in 1 min, and blood was subsequently sampled (30 μl) through tail bleeding at t = -5 (pre), 2, 5, 10, 15, 20, and 30 min after start of the tracer infusion. Circulating plasma was derived from the dilution of fluorescein-labelled red blood cells (RBCs) and large vessel hematocrit [22,25,27]. Thereto, blood (~1 ml) was collected from a donor mouse (C57Bl/6) by cardiac punction, centrifuged, and the RBCs labelled with sodium fluorescein (250 mg/ml) for 5 min. After washing, the labelled cells were resuspended in saline to the initial volume; two min before infusion, 0.1 ml of the labelled blood was mixed with an equal volume of Dex40 (15 mg/ml) and 0.1 ml of this tracer mix was infused in the animal in 1 min. Blood samples (5 μl) were collected in heparinized capillaries through tail bleeding at t = -5 (pre), and 2, 3, 4, and 5 min after start of the infusion for determination of the fraction of labelled RBCs, while in addition 30 μl samples were collected in ~30 s at t = -5 (pre), 2, 5, 10, 15, 20, and 30 min for determination of Dex40 concentrations.

In each mouse, urine production was assessed by collecting visibly excreted urine during the duration of the experiment in a capillary tube together with the remaining urine content in the bladder right after the mouse had been euthanized (at t = 35 min) [24,25]. In addition, the heart and the kidneys were collected after the experiment, blotted, and their wet weight measured [24]. Tissues were stored at 70°C for three days, and then weighed again for obtaining dry weight.

### Tracer analysis

Labeled RBCs were measured using a FACScan analyzer (FACSCalibur; Becton Dickinson, Mountain View, CA), with at least 100,000 cells being counted to measure the circulating fraction of labeled cells [22,25,27]. Data were analyzed using Cellquest (Becton Dickinson, San Jose, CA). The circulating plasma volume was calculated as [(1 – *Ht*) × *Vrbc*] / *Ht*, where *Vrbc* is the circulating red blood cell volume ([1/circulating fraction of labelled RBCs] × volume of labelled cells injected) and *Ht* is the large vessel hematocrit [22,25,27]. The fraction of labeled cells at t = 2, 3, 4, 5 min was averaged and used as circulating fraction; unlabeled erythrocytes obtained before the injection (t = -5 min) served as negative controls. The sum of both circulating RBC and plasma volume revealed total blood volume.

For the blood samples containing dextrans, capillaries were centrifuged, hematocrit was determined, and plasma collected and stored at -20°C until fluorescence analysis. In each sample, fluorescence was measured at 490/535 nm (excitation/emission) for the Dex70 and at 595/615 nm for Dex40 with a spectrophotometer (VICTOR; PerkinElmer) and dextran concentrations calculated in reference to defined dilutions of the infused tracer mix in plasma from donor mice [24-27]. Concentrations were normalized to the amount injected. For Dex70, the time-concentration curve was fitted with a mono-exponential function [24-27], and the initial distribution volume determined from the extrapolated dilution at the start of tracer injection. For Dex40, linear extrapolation of the concentration between t = 2 and t = 5 min to the start of injection was used, because this dextran has been indicated to rapidly egress from the circulation [25]. Vascular clearance was defined as the percentage decrease in dextran concentration at the end of the experiment (t = 30 min) compared to the extrapolated concentration at the start of tracer injection (t = 0 min) [25,26].

Urine samples were stored at -20°C until analysis when dextran concentrations were calculated in reference to defined dilutions of the infused tracer mix in urine from donor mice. The percentage dextran recovery in the urine in an experiment was determined from the total volume of urine sampled and its dextran concentration, normalized to the amount injected [25].

### Statistics

Summary data are reported as means ± SEM. Because of the differences in body weight between db/db and C57Bl/6 mice, tracer distribution volumes were normalized for body weight. Differences between the metformin-treated mice and control db/db mice, as well as the reference C57Bl/6 mice, were evaluated using unpaired t-tests. Results were considered statistically significant with *P* ≤ 0.05.

## Results

**Figure 1:**
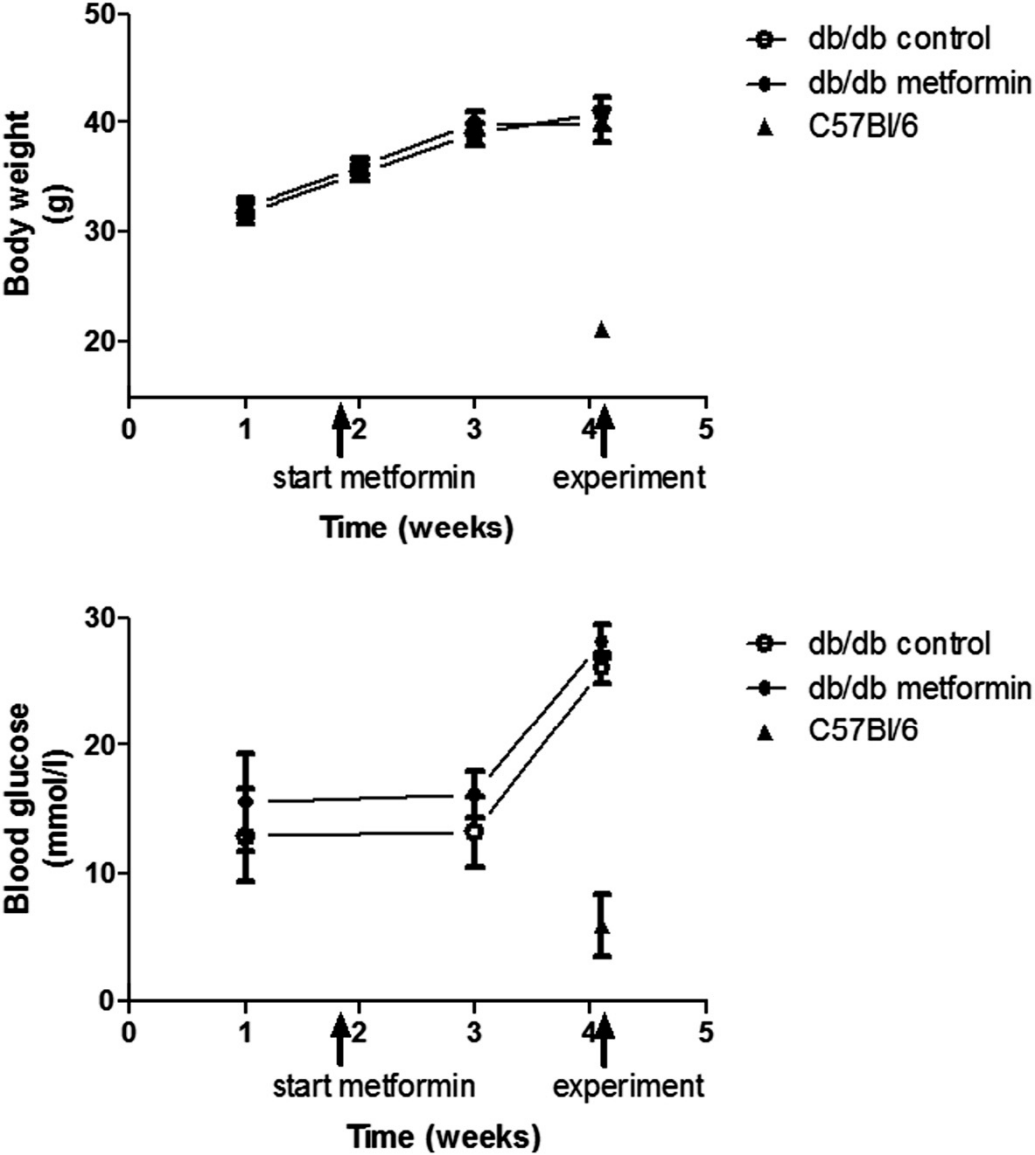
**Body weights and blood glucose levels.** Top: body weight was measured every week in the db/db mice (n = 20). The first measurement (week 1) was at an age of 6 weeks. One week later the control group (n = 10) continued with normal drinking water while the experimental group (n = 10) started with the metformin in the water for two weeks until the acute experiment (week 4). C57Bl/6 mice (n = 10) were used as reference group in the acute experiment. Bottom: blood glucose in the db/db mice was measured one week before and one week after start of the metformin, as well as during the acute experiment. The sharp rise in glucose during the acute experiment is likely explained by the anesthetic conditions.

At an age of 7 weeks, db/db mice were divided in a control group which continued with normal drinking water for two weeks and an experimental group which received metformin at a concentration of 0.33 mg/ml in the drinking water for the same period. Daily water intake was 32.4 ± 2.5 and 37.7 ± 0.9 ml/100 g body weight in the control and metformin-treated db/db mice, respectively (P = 0.08). Body weight and fasting blood glucose were weekly measured, and were not affected by the metformin (Figure 1).

**Figure 2:**
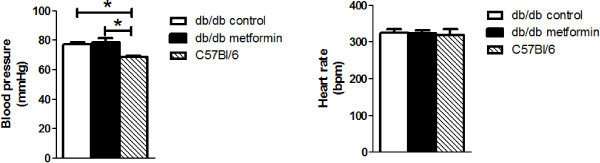
**Hemodynamics.** Carotid artery blood pressure (left) and heart rate (right) were measured continuously in the anesthetized mice during the acute experiment. Values represent average pressure/rate for the entire duration of the experiment (from t = -10 min until t = 30 min). *, P < 0.05 versus C57Bl/6 mice.

After two weeks, glycocalyx barrier properties were measured in an acute experiment. Blood glucose during the experiment was found to be similarly elevated in both groups of db/db mice compared to the earlier measurements in the awake animals (Figure 1), likely as a result of the anesthesia. While blood pressures were higher in the db/db mice compared to the C57Bl/6 mice during the experiment, they did not differ between the metformin and control group (Figure 2). Heart rates were comparable between the three groups of mice (Figure 2).

**Figure 3:**
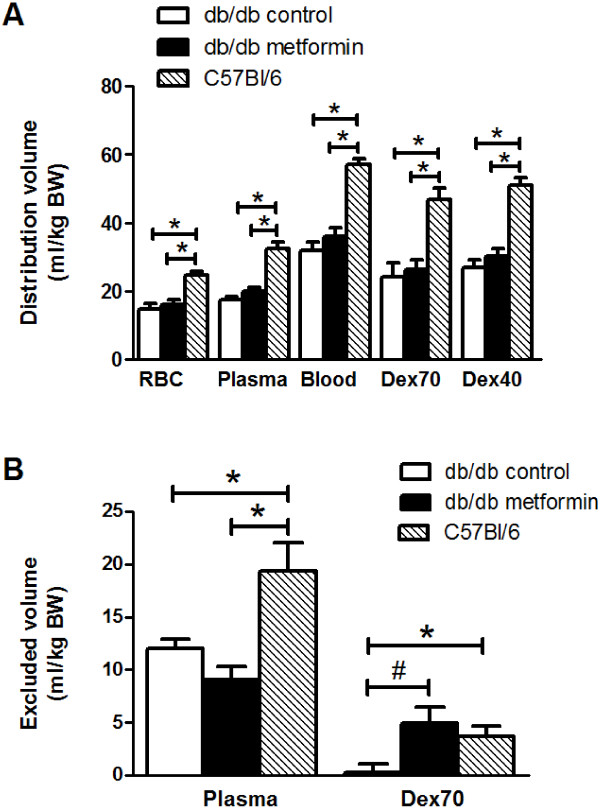
**Systemic (glycocalyx excluded) distribution volumes. A**: systemic volumes of distribution (normalized to body weight) of circulating blood, i.e., the sum of red blood cell (RBC) and plasma volume (derived from RBC space and hematocrit), and 70 (Dex70) (both n = 5 per group) and 40 kDa dextrans (Dex40) (n = 10 per group). *, P < 0.05 versus C57Bl/6 mice. **B**: whole-body glycocalyx volumes for circulating plasma and Dex70 (both n = 5 per group), calculated from the difference between their respective distribution volume versus that of Dex40. *, P < 0.05 versus C57Bl/6 mice; #, P < 0.05 metformin versus control db/db.

Whole-body distribution volumes (normalized to body weight) of the different tracers are shown in Figure 3A. Hematocrits (in %) were not different between the three groups (control db/db: 45.8 ± 2.8; metformin db/db: 45.0 ± 3.1; C57Bl/6: 44.0 ± 3.8). As shown in our previous studies [25,27], distribution volumes were lower for circulating plasma, derived from the dilution of labeled RBCs and hematocrit, compared to the dextrans (Figure 3A). Distribution volumes for all considered tracers were lower in the db/db compared to the C57Bl/6, and did not statistically differ between the metformin treated and control db/db groups (Figure 3A). Glycocalyx-excluded volumes for plasma and Dex70, derived from the individual differences in Dex40 distribution volume versus those for circulating plasma and Dex70, respectively, are shown in Figure 3B. In line with our previous study [25], exclusion volume for plasma was larger than for Dex70 in all animal groups. While exclusion for plasma was not affected in the metformin treated animals, exclusion volume for Dex70 was significantly improved in this group, and not different from the C57Bl/6 animals anymore.

**Figure 4:**
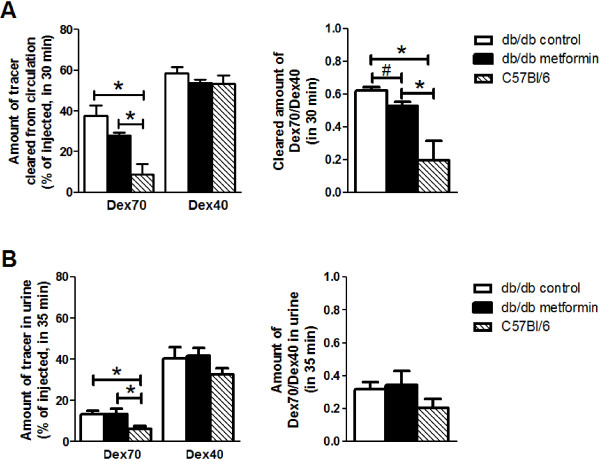
**Vascular clearance and urine appearance of dextrans. A**. Left. Calculated amount of 70 (Dex70) and 40 kDa dextran (Dex40) cleared from the circulation after injection (t = 0 min) until the last time point of blood sampling (t = 30 min). Right: cleared amount of Dex70 divided by Dex40. *, P < 0.05 versus C57Bl/6 mice; #, P < 0.05 metformin versus control db/db. **B**. Left. Measured amount of dextran in urine collected during the experiment and in bladder after euthanization of the animal (t = 35 min). Right: urine amount of Dex70 divided by Dex40. *, P < 0.05 versus C57Bl/6 mice.

Dextran clearance data are shown in Figure 4. In line with previous studies [24-26], vascular clearance of Dex40 was larger than that of Dex70, yet was not different between the three animal groups. Vascular clearance of Dex70 was higher in the db/db mice compared to the C57Bl/6 mice, and tended to decrease after treatment with metformin (38 ± 5% of injected Dex70 cleared in 30 min in control db/db versus 28 ± 1% in metformin treated group (p = 0.086)) (Figure 4A, left). When accounting for individual differences in Dex40 clearance rate [26], Dex70 clearance was significantly decreased in the metformin treated db/db compared to the control db/db mice, but still larger than in the C57Bl/6 mice (Figure 4A, right).

The diminished vascular clearance of Dex70 compared to Dex40 in the metformin treated mice appeared not to be associated with a reduction in the amount of Dex70 excreted in the urine at the end of the experiment. Thus, about 15% of the injected amount of Dex70 was detected in the urine of both groups of db/db mice, which was about twice (P < 0.05) of that found in the urine of the C57Bl/6 mice (Figure 4B, left). Similar to the vascular clearance characteristics, the amount of Dex40 retrieved in urine was higher than that of Dex70, i.e., about 35-40% of the injected amount, and comparable for all three animal groups (Figure 4B, left). When correcting for differences in Dex40 recovery between individual animals, no differences between groups were found in the amount of Dex70 retrieved in urine at the end of the experiment.

**Figure 5:**
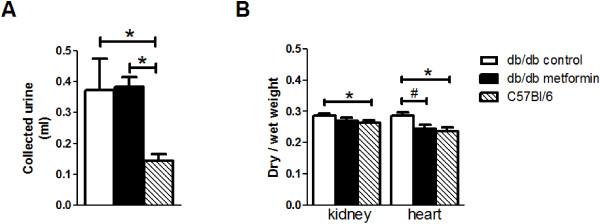
**Urine production and tissue hydration. A**. Urine volumes collected during the experiment and in bladder after euthanization of the animal (t = 35 min). *, P < 0.05 versus C57Bl/6 mice. **B**. Dry-to-wet weight ratios. Heart and kidneys were collected after the experiment and their wet weight measured, after which organs were stored at 70°C for three days, and then weighed again for obtaining dry weight. *, P < 0.05 versus C57Bl/6 mice; #, P < 0.05 metformin versus control db/db.

The collected urine volumes were not different between the control and metformin treated db/db mice, and both were larger than in the C57Bl/6 mice (Figure 5A). To evaluate organ hydration, the dry-to-wet weight ratio for heart and kidneys were determined [24]. Higher dry-to-wet weight ratio’s, reflecting decreased water content, were found for both tissues of the control db/db mice versus C57Bl/6 mice. In contrast, the ratios of the metformin treated db/db mice were not different from those of the C57Bl/6 mice (Figure 5B), and hydration of the heart was significantly improved in the metformin treated db/db mice versus the control db/db mice.

## Discussion

In the present study we showed that metformin treatment for 2 weeks in db/db mice resulted in an improvement of the endothelial glycocalyx barrier properties in the circulation as evidenced by the increased whole-body exclusion volume of Dex70 compared to Dex40 and enhanced vascular retention of Dex70 compared to Dex40. Further, hydration of heart and kidney was improved by metformin. The two week treatment with metformin did not significantly affect blood glucose levels, indicating that the effects of metformin occurred independent from a change in fasting glucose. These data suggest that the previous reported cardiovascular benefits of metformin may involve an improvement of the endothelial glycocalyx.

### Short-term metformin does not affect blood glucose

Metformin has been famed for its protective effect against vascular complications in NIDDM independent of its glycemic lowering action [6,7]. Improvement of endothelial function has been considered to contribute to metformin’s vasculoprotective effect [11]. Metformin treatment was for example shown to improve endothelium-dependent vasodilation and microvascular reactivity in humans with the metabolic syndrome [8,29], and to improve arterial stiffness in patients with nonalcoholic fatty liver disease, which was partly explained by an increase in circulating adiponectin levels [30]. Furthermore, a recent randomized controlled trial with a follow-up for more than four years in patients with insulin-treated NIDDM, showed improved circulating levels of vWf and sVCAM-1 with metformin treatment, indicating specific effects of this drug on endothelial function [31]. Hereto related, direct beneficial effects of metformin on human coronary artery endothelial cell viability, regeneration, and apoptosis have been recently reported [32]. Altogether, these data add another valuable property to metformin’s therapeutic effect, advancing its clinical utility in patients with NIDDM in whom endothelial dysfunction is prominent.

Because the endothelial glycocalyx has been indicated to play a major role in protection of the endothelium and regulation of its many important functions [13-15], we were interested in the current study whether metformin would improve the recently reported glycocalyx perturbation in db/db mice [24]. Because changes in blood glucose itself have been shown to affect glycocalyx properties [21,24], we aimed in the present study to limit the blood-glucose lowering effect of metformin by treating the mice for a short period of time only at a relatively mild dose of the drug. Indeed, we did not observe a reduction in blood glucose levels in the metformin treated db/db mice compared to the control db/db mice. Water intake during metformin administration was ~35 ml/100 g/day (see Results), which with the dose of 0.33 mg/ml agrees with an average metformin intake of ~100 mg/kg per day. In previous studies in mice, it was shown that higher doses of metformin (150–250 mg/kg/day) for a longer period of time (4 weeks) did lower blood glucose levels [33,34]. Also, contrasting to previous studies which showed that a 3 week treatment with metformin at a dose of 300–500 mg/kg/day prevented the hypertension induced by fructose feeding in rats [11,35], the two week treatment period of the lower dose in the current study did not affect blood pressure in the db/db mice.

### Measurement of glycocalyx barrier properties: methodological considerations

**Figure 6:**
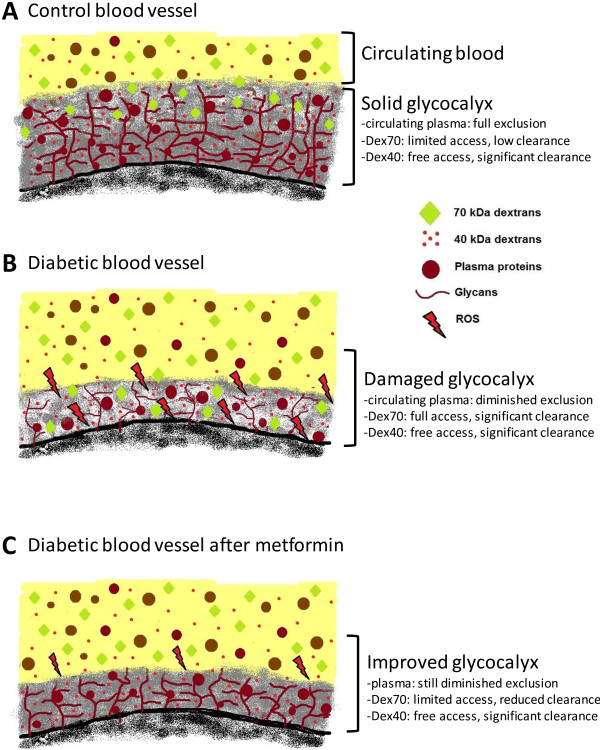
**Schematic illustration of the hypothesized effect of metformin on the glycocalyx barrier.** The endothelial glycocalyx is considered to form a highly hydrated mesh of polysaccharide structures and adsorbed plasma proteins on the luminal side of all blood vessels. **A**. Under normal physiological conditions, the structure of the glycocalyx layer is stable and its molecular composition represents a dynamic balance between continued biosynthesis versus shedding of glycans in combination with the absorption and release of plasma proteins. A healthy glycocalyx has been shown to constitute a significant barrier for the circulating blood. While its sieving properties hinder the access of 70 kDa dextrans (Dex70), 40 kDa dextrans (Dex40) appear to have unlimited access to the entire glycocalyx domain. **B**. The balance between glycocalyx synthesis and breakdown is, however, disrupted in diabetes. This results in an impairment of the barrier properties of the glycocalyx, causing a diminished exclusion of the circulating blood and permeation of Dex70. Reactive oxygen species (ROS) are indicated to be involved in the glycocalyx breakdown. **C**. Two weeks of metformin treatment is associated with a partial recovery of the diabetes-associated disrupted glycocalyx barrier. While exclusion of circulating plasma is still impaired, the exclusion of Dex70 is significantly increased. As a result, the access of Dex70 into the glycocalyx is hindered and Dex70 stays mainly confined to the circulation. While the mechanisms underlying the glycocalyx improvement by metformin remain to be elucidated, alleviation of oxidative stress is suggested to play a role. Diagrams are not drawn to scale.

Figure 6 shows a schematic illustration of the proposed interpretation of the systemic glycocalyx measurements in the current study. The methodology for delineating glycocalyx barrier properties, which was used in the current and previous studies [21,24-27], is based on the tracer dilution technique, with the choice of tracers being inferred from intravital microscopic observations of intravascular tracer distribution in cremaster capillaries [36-38] and in mesenteric blood vessels [39,40]. These observations revealed that a healthy glycocalyx excludes circulating blood and constitutes a significant barrier for FITC-labeled dextrans of 70 kDa and larger, without apparent hindrance of Texas Red-labeled 40 kDa dextrans (Figure 6, top panel). A severely damaged glycocalyx, such as after light-dye treatment, is, however, associated with the access of the larger dextrans into the entire glycocalyx domain and subsequent transvascular escape [28,39] (Figure 6, middle panel). These sieving features of the glycocalyx were more recently applied to derive estimates of systemic glycocalyx volume and barrier properties in experimental animals [24-27,41] and in humans [13,22], and to study the effect of various cardiovascular risk factors on the glycocalyx. In hamster and mice, Dex70 has been primarily used as circulating intravascular tracer and, agreeing with the intravital microscopy studies, its whole-body distribution volume was smaller than that of Dex40 [24-26,41]; moreover, in line with their significant exclusion by the glycocalyx, Dex70 was hardly cleared from the circulation while there was a considerable loss of Dex40 in time. A similar behavior of Dex70 versus Dex40 was also observed in the C57Bl/6 group in the current study (Figures 3 and 4). Since both systemic distribution volume and vascular clearance of Dex70 were subsequently found to move towards those of Dex40 during enzymatic challenge of the glycocalyx [25,26], it is pointed out that both a reduction in Dex70 exclusion volume and an increased Dex70 clearance are indicative of a compromised glycocalyx. One should keep in mind, however, that an increased Dex70 clearance may involve additional mechanisms determining vascular permeability than the increase in permeability of the glycocalyx alone.

The observation that Dex70 and Dex40 distribution volumes were near equal in db/db mice in the current study thus suggests the presence of a compromised glycocalyx barrier which has lost its size-selectivity in this mouse model (Figure 6, middle panel), which is in line with previous experimental data showing a detrimental effect of hyperglycemia on the glycocalyx [21,22,24]. The impaired exclusion of Dex70 was associated with a significant vascular clearance of Dex70 at a rate that was comparable to that observed after enzymatic glycocalyx degradation [25,26]. In contrast, the clearance of Dex40 was not affected but still higher than that of Dex70. This relative insensitivity of Dex40 clearance to glycocalyx damage was demonstrated in the previous animal studies as well [24-26], and supports the suggestion that this dextran seems not hindered by the glycocalyx barrier. Further, Dex40 clearance seems to be governed largely by the kidneys, as exemplified by the retrieval of ~60-70% of the cleared amount in the urine in the current and our previous study [25]. In contrast, the amount of Dex70 retrieved in the urine was much lower, also in the db/db mice, indicating that other mechanisms than renal excretion may underlie the increased vascular clearance of Dex70 in the presence of a compromised glycocalyx barrier. A likely mechanism may be transvascular leakage of Dex70 due to the impaired glycocalyx barrier properties in combination with enlarged interendothelial gap widths or a damaged endothelial plasma membrane; however, measurements of dextran extravasation are needed for a correct interpretation of this.

In addition to Dex70, we evaluated the glycocalyx barrier properties by assessing the differential distribution of circulating plasma versus that of Dex40, an approach which has been applied in the past as well [21,22,25,27]. In line with our previous study [25], we found that the circulating plasma volume was smaller, and hence its glycocalyx excluded volume was larger, than for Dex70 (Figure 3), reflecting the fact that these dextrans seem to have partial access into the glycocalyx domain compared to the circulating plasma (Figure 6, top panel). The db/db mice showed a ~50% decrease in the plasma-excluded glycocalyx volume (Figure 6, middle panel), which is comparable to the effect of acute enzymatic breakdown on this parameter [25], and supports the Dex70 data that the glycocalyx was significantly compromised in these mice. It has been indicated in the past that most likely reactive oxygen species are involved in the glycocalyx degradation in diabetes [21,24], because of their abundance during hyperglycemic conditions [42,43] and the recurring observation that the glycocalyx is particularly vulnerable to free radical damage [15,28,44,45].

### Short-term metformin improves glycocalyx barrier properties and organ hydration

The dichotomy between the measurements of the glycocalyx barrier properties based on circulating plasma versus Dex70 as glycocalyx-excluded tracer was exemplified by the results of the two weeks of metformin treatment in the db/db mice. Whereas the treatment did not improve the glycocalyx exclusion of circulation plasma, it robustly alleviated the compromised glycocalyx barrier for Dex70 (Figure 6, lower panel), as evidenced by the increased Dex70 excluding glycocalyx volume which appeared indistinguishable from that measured in the C57Bl/6 mice (Figure 3). The improvement coincided with a modest reduction in the vascular clearance of Dex70 (Figure 4). This reduced Dex70 clearance from the circulation might represent a slow equilibration of Dex70 with the glycocalyx domain and their subsequent extravasation [25]. The enhanced exclusion and retention of Dex70 seems not explained by a potential binding of the dextran to the residing metformin since this would be expected to lower circulating dextran concentrations, thereby resulting in an overestimation rather than a reduction of their distribution volume and clearance. Also, Dex40 distribution and clearance were not different in the metformin treated mice compared to the non-treated ones (Figure 4), making the possibility of dextran-binding by metformin very unlikely. The data of the current study, therefore, indicate that two weeks of metformin were associated with significant (i.e., restored Dex70 exclusion) but not complete improvement (i.e., no alleviation of plasma exclusion) of the glycocalyx barrier properties in the db/db mice. This incomplete recovery can be attributed to the relative short treatment duration and remaining hyperglycemia and it would be interesting to examine in future studies the effect of metformin on recovery of the glycocalyx for longer treatment periods and in the presence of lower blood glucose levels. Moreover, in the current study the direct physiological impact of the metformin-induced improvement of the glycocalyx barrier could not be readily appreciated. While metformin treatment for several weeks has been associated with improved vascular permeability in NIDDM and cyclic edema [12,46], the improvement of vascular Dex70 retention in the current study was modest after the two weeks of metformin. Thus, the resultant clearance in the metformin treated mice in the current study was still ~30% and much higher than the minimal (~10%) clearance of Dex70 in the C57Bl/6 mice (Figure 4). Also, renal clearance of Dex70 seemed not to be improved in the db/db mice. This may contrast to some previous studies showing alleviation of microalbuminuria by metformin in diabetes patients and animal models [12,47], although a beneficial effect of metformin on albumin excretion has not been consistently demonstrated [31]. As a consequence, the contribution of the improved glycocalyx barrier for Dex70 to the increased organ hydration in the current study remains uncertain. Thus, while two weeks of metformin treatment in the db/db mice resulted in comparable dry-to-wet weight ratios in heart and kidney versus the C57Bl/6 mice, hematocrit was not decreased in the metformin treated animals, indicating that intravascular fluid retention was not augmented. It was remarkable, though, that, contrasting to our previous study [24], hematocrit in the control db/db mice was not elevated to begin with. On the other hand, while there was a trend for a higher daily water intake in the metformin group (P < 0.08), urine volumes collected in the acute experiments were very comparable for both diabetic groups; also, whole-body volumes of distribution for the different tracers did appear to be somewhat larger in the metformin treated mice compared to the controls (Figure 3), suggesting that metformin increased the tracer available microvascular volume either in a structural or functional manner. In line herewith, metformin has previously been shown to improve obesity- and diabetes-induced reductions in microvascular and capillary density [12]. Again, the seemingly marginal effects of the two weeks of metformin on intravascular fluid retention may be contributed to the relatively short metformin treatment and persistent hyperglycemia in the current experiments.

### Clinical relevance and possible mechanisms

Although the direct contribution of the improved glycocalyx barrier for vascular permeability could not be appreciated in the current study, the potential of alleviation of a perturbed glycocalyx as target for improving vascular permeability in diabetes was clearly indicated by a previous study of Broekhuizen and co-workers [23]. In this study, administration of sulodexide for 2 months in patients with NIDDM resulted in improved glycocalyx barrier dimensions in the sublingual and retinal microcirculation and this improvement was associated with an almost normalization of the transcapillary escape rate of albumin towards levels found in non-diabetic controls. While it is envisioned that treatment with sulodexide, which is a mixture of heparan and dermatan sulfates, may restore a damaged glycocalyx by supplementation of glycocalyx constituents, the mechanisms underlying the improved glycocalyx barrier properties by metfomin seem less straightforward. The most prominent components of the glycocalyx are the glycosaminoglycans heparan sulfate and chondroitin sulfate, which are sulfated and are linked to membrane bound proteoglycans, and hyaluronan [14,15,20,48] (Figure 6). In addition, adsorbed blood-borne soluble proteins comprise substantial components of the glycocalyx (Figure 6). Under normal physiological conditions, the structure of the glycocalyx layer is stable and its molecular composition represents a dynamic balance between continued biosynthesis versus shedding of glycans in combination with the absorption and release of plasma proteins. This balance is, however, easily disrupted as the labile nature of the glycocalyx has been demonstrated in several experimental studies, showing rapid shedding of components of the glycocalyx in response to, amongst others, inflammation, hyperglycemia, septic shock, and ischemia-reperfusion; in several cases a role for reactive oxygen species has been indicated in the detrimental effect [15,44,45,49]. The cellular signaling cascades initiating shedding of the glycocalyx are not fully understood, and may involve (matrix metallo) proteases and lyases synthetized by the endothelium [15]; in addition, pathological conditions may also affect the biosynthesis of new components, and interfere with the absorption of plasma proteins. Altogether, these data exemplify the complex and dynamic nature of the glycocalyx. As a result, it is challenging to indicate the underlying mechanisms responsible for the improved glycocalyx barrier for Dex70 in our study. Nevertheless, alleviation of oxidative stress associated with hyperglycemia and diabetes is likely an important contributor [43]. Metformin has been demonstrated to have the potency to decrease oxidative stress by impairing the production of reactive oxygen species in vascular cells [50,51] and reducing the formation of pro-oxidative advanced glycation endproducts [12,52]. In addition, metformin may protect the vasculature from oxidative stress by impairing the glycation of the “natural” anti-oxidants albumin [52,53] and superoxide dismutase [12]. These proteins are under normal conditions bound within the glycocalyx, and it is well conceivable that by inhibiting their glycation metformin permits an improved interaction of these proteins with the glycocalyx structures. The ultimate outcome of these and potential other actions of metformin is considered to shift the disrupted balance between glycocalyx production and breakdown towards a net build-up of an effective glycocalyx barrier (Figure 6, bottom panel).

## Conclusions

Metformin has in the past been associated with less cardiovascular morbidity and mortality in patients with NIDDM; this effect was shown to be in part independent of an improvement in glycemic control. In the current study it was found that two weeks of metformin treatment in db/db mice was associated with significant recovery of the endothelial glycocalyx barrier without a change in blood glucose levels. Given the indicated important role of the glycocalyx as orchestrator of vascular homeostasis and its close association with a firm endothelial function, our results imply that an improvement of endothelial glycocalyx function may contribute to the suggested cardiovascular benefits of metformin. Alleviation of glycocalyx damage is proposed a useful therapeutic target in NIDDM.

## Abbreviations

NIDDM: Non-insulin dependent diabetes mellitus; RBC: Red blood cell; Dex70: 70 kDa dextrans; Dex40: 40 kDa dextrans; NO: Nitric oxide; Ht: Hematocrit; Vrbc: Circulating red blood cell volume; vWf: Von Willebrand factor; sVCAM-1: Soluble vascular cell adhesion molecule 1; ROS: Reactive oxygen species.

## Competing interests

The author(s) declare that they have no competing interests.

## Authors’ contributions

BJM carried out the experiments and drafted the manuscript. CJZ participated in the design of the study. JvH participated in the design of the study. HV participated in the design of the study. JWGEvT conceived of the study, and participated in the design and coordination of the study, and drafted the manuscript. All authors read and approved the final manuscript.
